# Best Practices and Barriers to Obesity Prevention in Head Start: Differences Between Director and Teacher Perceptions

**DOI:** 10.5888/pcd14.170297

**Published:** 2017-12-21

**Authors:** Courtney Byrd-Williams, Erin E. Dooley, Shreela V. Sharma, Ru-Jye Chuang, Nancy Butte, Deanna M. Hoelscher

**Affiliations:** 1 Michael & Susan Dell Center for Healthy Living at The University of Texas Health Science Center at Houston (UTHealth), School of Public Health in Austin, Austin, Texas; 2 Michael & Susan Dell Center for Healthy Living at The University of Texas Health Science Center at Houston (UTHealth) School of Public Health in Houston, Houston, Texas; 3 US Department of Agriculture, Agricultural Research Service Children’s Nutrition Research Center, Department of Pediatrics, Baylor College of Medicine, Houston, Texas

## Abstract

**Introduction:**

Practices and barriers to promoting healthy eating and physical activity at Head Start centers may influence children’s energy balance behaviors. We examined differences between directors’ and teachers’ perspectives on best practices and barriers to promoting healthy eating and physical activity in Head Start centers.

**Methods:**

We conducted a cross-sectional study of directors (n = 23) and teachers (n = 113) at 23 Head Start centers participating in the baseline assessment of the Texas Childhood Obesity Research Demonstration study. Participants completed surveys about practices and barriers to promoting healthy eating and physical activity. Multilevel regression models examined differences between director and teacher responses.

**Results:**

More than half of directors and teachers reported meeting most best practices related to nutrition and physical activity; few directors or teachers (<25%) reported conducting physical activity for more than 60 minutes a day, and less than 40% of teachers helped children attend to satiety cues. Significantly more directors than teachers reported meeting 2 nutrition-related best practices: “Teachers rarely eat less healthy foods (especially sweets, salty snacks, and sugary drinks) in front of children” and “Teachers talk to children about trying/enjoying new foods” (*P* < .05). No barrier to healthy eating or physical activity was reported by more than 25% of directors or teachers. Significantly more teachers than directors reported barriers to healthy eating, citing lack of food service staff support, limited time, and insufficient funds (*P* < .05).

**Conclusion:**

More barriers to healthy eating were reported than were barriers to physical activity indicating that more support may be needed for healthy eating. Differences between responses of directors and teachers may have implications for future assessments of implementation of best practices and barriers to implementation related to nutrition and physical activity in early care and education centers.

## Introduction

Head Start, the largest federally funded early care and education (ECE) program for preschoolers in the United States, provides comprehensive education, social, and health services to low-income families. Approximately one-third of children who attend Head Start centers are overweight or obese (1–3). As part of health services, Head Start providers are required to incorporate efforts to combat childhood obesity in their programs.

Many studies have been conducted in the ECE setting to examine the effect of an intervention program on obesity in early childhood (4). To understand an intervention’s effects on childhood obesity requires an understanding of how employees perceive the degree to which best practices are being met at ECE centers and the barriers they perceive to promoting preschoolers’ energy balance behaviors.

Research has documented barriers to implementing obesity prevention efforts at ECE centers, including Head Start centers. In a national study, Head Start directors reported that primary barriers are lack of staff time, resources, and knowledge about nutrition and physical activity (5). Results from several statewide surveys of ECE centers indicate similar barriers to promoting healthy eating and physical activity (6,7). However, studies were conducted at the center level and did not include classroom-level or teacher-reported attitudes or practices.

Teachers are generally responsible for implementing obesity prevention efforts in the ECE center (4). As such, it is important to understand the perspectives of Head Start teachers. Therefore, the purpose of this study was twofold: 1) to describe director-reported and teacher-reported best practices and perceived barriers to promoting nutrition and physical activity in Head Start centers and 2) to examine differences in perceptions as reported by directors and teachers.

## Methods

The data for this cross-sectional, exploratory analysis was collected as part of the baseline assessment for the Texas Childhood Obesity Research Demonstration (TX CORD) study, a multilevel intervention to address childhood obesity in low-income populations (8). The methods for data collection of TX CORD have been reported elsewhere (8,9). Before the implementation of TX CORD, Head Start center managers (hereinafter directors) and classroom teachers at 23 Head Start centers in Austin, Texas, and Houston, Texas, were surveyed in the summer and fall of 2012. Study staff members distributed and collected survey materials from center directors and teachers. Surveys examined the center’s current practice regarding adherence to best practice standards around nutrition and physical activity and the current barriers faced by the Head Start center staff. The institutional review boards at The University of Texas Health Science Center–Houston and Baylor College of Medicine approved all protocols and procedures.

Directors (n = 23) and teachers (n = 113) were recruited from the 23 Head Start centers participating in TX CORD. Inclusion criteria were that the respondent be employed at the Head Start center and be able to complete the survey in English. All those approached met both inclusion criteria. Directors and teachers completed written surveys that consisted of questions from the Nutrition and Physical Activity Self-Assessment for Child Care (NAP SACC) (10) and The Child Care Nutrition and Physical Activity Assessment Survey (11). NAP SACC has been used to survey both directors and teachers (12). The original NAP SACC questions were used to assess current nutrition-related and physical activity-related behaviors. Each question had 4 unique, practice-specific response options, including the best practice for that item. NAP SACC questions were dichotomized into whether the best practice was reported as met or not. A best practice recommendation was reported as met if selected and as not met if any of the other 3 response options were selected. Questions about perceived barriers to promoting healthy eating and physical activity in child care centers were adapted from The Child Care Nutrition and Physical Activity Assessment Survey (11) to examine healthy eating and physical activity elements separately. For example, the original survey question asked “Are any of the following barriers to promoting healthy eating and physical activity practices in your center?” with a check-all-that-apply response option. Our study examined healthy eating and physical activity separately without modifying the response options (ie, “Are any of the following barriers to promoting healthy eating practices in your center?” and “Are any of the following barriers to promoting physical activity practices in your center?”). Response options to the barrier questions were dichotomous (yes/no).

Descriptive analyses were conducted on survey responses. We computed means, frequencies, and percentages of director and teacher responses and difference in percentages between director and teacher responses. To account for nesting, multilevel regression models were used to examine differences between director and teacher responses. Differences were considered significant at *P* < .05. All statistical analyses were performed using SPSS version 24.0 (IBM Corp) and SAS version 9.4 (SAS Institute, Inc).

## Results

Directors and teachers were predominantly female (96%) and considered English their primary language (>90%), and about 40% were Hispanic (Table 1). Directors and teachers had a mean age of 51 years (standard deviation [SD], 8 y) and 43 years (SD, 11 y), respectively, and more than half were college graduates (78% and 57%, respectively). More than 70% of both directors and teachers reported that several nutrition and physical activity best practices were being implemented. 

**Table 1:** Baseline Characteristics, Directors and Teachers (N = 136) in Head Start Centers Participating in the TX CORD Study, Texas 2013

Characteristic	Directors (n = 23)^a^	Teachers (n = 113)^a^
**Age, mean (standard deviation), y**	50.8 (8.0)	42.5 (11.2)
**Sex (female)**	95.7 (22)	96.0 (105)
**Primary language**		
Speak English only	60.9 (14)	46.8 (51)
Speak more English than another language	8.7 (2)	18.4 (20)
Speak both English and another language, equally	30.4 (7)	25.7 (28)
Speak another language more than English	0	9.2 (10)
Speak only another language	0	0
**Highest grade completed**		
High school graduate	4.4 (1)	11.0 (12)
Some college or technical school	17.4 (4)	32.1 (35)
College graduate	78.3 (18)	56.9 (62)
**Race/ethnicity**		
Non-Hispanic white	4.3 (1)	3.7 (4)
Hispanic or Latino	39.1 (9)	44.0 (48)
Black or African-American	52.2 (12)	48.6 (53)
Other	4.3 (1)	3.7 (4)

Abbreviation: TX CORD, Texas Childhood Obesity Research Demonstration.

a Values are % (no.) unless otherwise indicated.

Of the 7 nutrition-related best practices, we found no significant differences in the number of best practices that directors (mean, 4.65; SD, 0.88) and teachers (mean, 4.15; SD, 1.72) reported meeting (*P* = .12). Overall, more than 60% of directors and teachers reported meeting nutrition-related best practices except for those related to attending to satiety cues (Table 2). Significantly more directors than teachers reported meeting 2 specific nutrition-related best practices: 1) “Teachers rarely eat less healthy foods (especially sweets, salty snacks, and sugary drinks) in front of children” (100% vs 69%, *P* = .001); and 2) “Teachers talk to children about trying and enjoying new foods” (87% vs 63%, *P* = .02). No significant differences were found between director and teacher responses for meeting the other 5 nutrition-related best practices (Table 2).

**Table 2:** Nutrition-Related and Physical Activity–Related Best Practices in Head Start Classrooms Reported by Head Start Directors and Teachers (N = 136), TX CORD Study, Texas 2013

Best Practice	Directors (n = 23)^a^, % (no.)	Teachers^a^^,^ b, % (no.)	Difference in Percentage^c^	*P *Value^d^
**Nutrition**				
Food is rarely or never used to reward a desired behavior	95.7 (22)	93.7 (104)	2.0	.64
Children who are picky eaters (able to eat food but resisting) are encouraged to try a new less favorite food	65.2 (15)	60.7 (68)	4.5	.69
When children eat less than half of a meal or snack, the teachers help determine if they are full before removing the plate	39.1 (9)	32.7 (36)	6.4	.56
Teachers talk with children about trying and enjoying healthy food	87.0 (20)	63.4 (71)	23.6	.02
Teachers rarely or never eat or drink less healthy foods (especially sweets, salty snacks, and sugary drinks) in front of the children	100.0 (23)	68.8 (77)	31.3	.001
When children request seconds, teachers help determine if they are still hungry before serving the requested food	17.4 (4)	19.4 (21)	−2.1	.85
Teachers or staff provide nutrition education for children 1 or more times per week	60.9 (14)	78.2 (86)	−17.3	.08
**Physical activity**				
Children are allowed to watch videos, television, or play computer games 1 time per week or less	59.1 (13)	58.2 (64)	0.9	.89
Teachers never restrict active play time for children who misbehave	91.3 (21)	78.9 (86)	12.4	.14
When weather is not suitable to go outdoors, indoor play space is available for all activities, including running	30.4 (7)	13.5 (15)	16.9	.05
During active (free) play time, teachers often or always join children in active play and make positive statements about the activity	65.2 (15)	70.3 (78)	−5.1	.62
Active (free) play time is provided to all children (including indoor and outdoor) more than 60 minutes each day	13.0 (3)	24.1 (27)	−11.1	.26
Teacher-led physical activity is incorporated into the curriculum 2 or more times each day	40.9 (9)	57.1 (64)	−16.2	.15

Abbreviation: TX CORD, Texas Childhood Obesity Research Demonstration.

a Each question had 4 unique response options that were specific to the item; one response was the best practice recommendation from the Nutrition and Physical Activity Self-Assessment for Child Care (NAP SACC) for that item (10). Answer options were then dichotomized by best practice recommendation being met if selected or not met if any of the 3 other response options were selected. The table provides the response of the best practice recommendation for each item.

b Teacher number varies from 108 to 112 because of missing data.

c Difference between number of director-reported and teacher-reported practices. A negative score indicates teachers are more likely to report a practice; a positive score indicates directors are more likely to report it.

d *P* values were calculated by using multilevel regression models to account for nesting of teachers within centers.

Of the 6 physical activity-related best practices, we found no significant differences in the number that directors (mean, 3.00; SD, 1.26) and teachers (mean, 3.01; SD, 1.34) reported being met (*P* = .12). Most directors and teachers reported that teachers never restrict activity for children who misbehave (>78%) and that teachers join children in active play and make positive statements about the activity (>65%, Table 2). Less than 25% of directors and teachers reported that teachers provide more than 60 minutes of active play each day. No significant differences were found in director and teacher responses for any physical activity–related best practices. (Table 2).

In response to the 14 nutrition-related barriers questions, teachers (mean, 1.56; SD, 2.28) reported significantly more barriers to promoting healthy eating than directors (mean, 0.39; SD, 1.47) (*P* = .001). Limited time and insufficient funds were the barriers to promoting healthy eating most frequently reported by both teachers and directors (Table 3). Significantly more teachers than directors reported 1) “Insufficient funds” (23% vs 4%, *P* = .02); 2) “Limited time for teaching nutrition” (23% vs 4%, *P* = .03); and 3) “Lack of support from food service staff” (17% vs 0%, *P* = .03). Among the other 11 perceived barriers to promoting healthy eating, no significant differences were found between teacher and director responses.

**Table 3:** Perceived Barriers to Promoting Healthy Eating and Physical Activity Reported by Head Start Directors and Teachers (N = 136), TX CORD Study, Texas 2013^a^

Barrier	Directors, n = 23, % (no.)	Teachers^b^, % (no.)	Difference in Percentage^c^	*P* Value^d^
**Do any of the following prevent you from promoting healthy eating at your center?**				
Serving unhealthy foods to children at center parties/social events	4.3 (1)	5.4 (6)	−1.1	.79
Lack of support from teachers	0	1.8 (2)	−1.8	.52
Lack of support from parents/families	8.7 (2)	13.6 (15)	−4.9	.52
Sales of unhealthy foods as fundraisers	0	5.4 (6)	−5.4	.26
Lack of training for food service staff	0	5.4 (6)	−5.4	.26
Lack of established policies on nutrition	0	6.4 (7)	−6.4	.21
Limitations of food service provider	4.3 (1)	11.6 (13)	−7.3	.27
Lack of support from administration	0	7.3 (8)	−7.3	.17
Lack of teacher training on nutrition education	4.3 (1)	12.6 (14)	−8.3	.25
Inadequate food preparation or storage facilities	4.3 (1)	13.5 (15)	−9.2	.12
Lack of nutrition education resources	4.3 (1)	15.3 (17)	−11.0	.06
Lack of support from food service staff	0	17.3 (19)	−17.3	.03
Limited time for teaching nutrition	4.3 (1)	23.4 (26)	−19.1	.03
Insufficient funds	4.3 (1)	23.4 (26)	−19.1	.02
**Do any of the following prevent you from promoting physical activity at your center?**				
Lack of support from administration	0	0	0	NA
Lack of teachers training on physical education	8.7 (2)	5.6 (6)	3.1	.56
Limited opportunities for physical education	8.7 (2)	5.6 (6)	3.1	.57
Lack of support from teachers	0	0.9 (1)	−0.9	.65
Lack of established policies on physical activity	4.3 (1)	6.4 (7)	−2.1	.59
Lack of support from parents/families	4.3 (1)	7.3 (8)	−3.0	.64
Insufficient funds	4.3 (1)	11.0 (12)	−6.7	.33
Lack of physical education resources	4.3 (1)	11.9 (13)	−7.6	.28

Abbreviation: NA, not applicable; TX CORD, Texas Childhood Obesity Research Demonstration.

a Answer options were yes or no.

b Teacher number varies from 108 to 112 because of missing data.

c Difference between number of director-reported and teacher-reported practice. A negative score indicates teachers are more likely to report the barrier; a positive score indicates directors are more likely to report it.

d *P* values were calculated by using multilevel regression models to account for nesting of teachers within centers.

In responses to the 8 questions about physical activity barriers, we found no significant differences in the number of barriers reported by teachers (mean, 0.46; SD, 0.94) and directors (mean, 0.34; SD, 1.11) (*P* = .59). Insufficient funds and lack of physical education resources were the most frequently reported perceived barriers to promoting physical activity reported by both teachers and directors. There were no significant differences between directors’ and teachers’ responses to any of the questions about perceived barriers to promoting physical activity (Table 3).

## Discussion

We examined differences in best practices and perceived barriers related to promoting healthy eating and physical activity at ECE centers. In general, directors and teachers reported meeting most best practices. Best practices to help children to attend to satiety cues and to provide adequate opportunities for physical activity were identified as areas needing improvement. We found significant differences between directors’ and teachers’ responses to several of the questions about best practices and perceived barriers. Teachers are generally responsible for the implementation of nutrition and physical activity interventions in the classroom; thus, differences between director and teacher responses on reports of practices within the center suggest that directors may not be fully aware of the need to improve the implementation of policies, programs, and best practices.

More than 70% of both directors and teachers reported that several nutrition and physical activity best practices were being implemented, and more than 93% of directors and teachers reported that food is never used as a reward. High implementation rates of the best practices are promising and have been seen in previous studies (6). One study of ECE programs in Minnesota and Wisconsin found 68% of ECE centers refrained from using food as a reward, and of those not meeting this best practice, 91% said it would not be difficult to implement the practice (6). In our study, more than 75% of directors and teachers endorsed the physical activity–related best practice that teachers never restrict active play time for children who misbehave. In the previously cited study (6), 66% of ECE centers did not restrict active play as punishment, and of those not implementing this practice, 83% reported that it would not be difficult to implement. The high rates of adherence to these best practices seen in this study and others and the perceived ease of their implementation suggest that they may be some of the easiest policies to implement at ECE centers.

ECE directors and teachers reported that the best practices least frequently met were helping children to attend to satiety cues and providing opportunities for physical activity. Attending to satiety cues, such as helping children determine if they are still hungry before serving requested seconds and helping children determine if they are full before removing their plate, are areas for improvement. In other Head Start centers, 50% (6) to 72% (5) of centers allowed children to decide when they were full during meal and snack times, suggesting that implementation of nutritional best practices across Head Start centers may vary. One of the physical activity practices rarely met according to both directors and teachers was providing more than 60 minutes of active play time. Although previous studies found that 59% (5) to 75% (6) of centers reported providing 60 minutes of daily activity, when physical activity was measured objectively, no centers were found to provide 60 minutes of moderate to vigorous physical activity per day (13). This suggests that teachers and directors need to be aware of the daily physical activity recommendations for early childhood and need to be mindful of the length and intensity of the activity provided. Future research studies should examine how to most effectively train caregivers so that they are aware of and able to implement best practices to promote healthy eating and physical activity.

In this study, directors were significantly more likely than teachers to respond that teachers rarely or never eat or drink less healthy foods, especially sweets, salty snacks, and sugary drinks, in front of children (100% vs 69%, respectively) and that teachers talk with children about trying and enjoying healthy food (87% vs 63%, respectively). Teachers’ behaviors and modeling regarding healthy and unhealthy foods are important, because caregivers’ practices have been found to affect children’s eating behaviors (14,15). Although most directors and teachers reported meeting these 2 best practices, which is consistent with the literature (16), directors were more likely to report the best practice as met, which may indicate their assessment of the ECE environment was not complete. When surveying the nutrition and physical activity environment of an ECE center, many studies solicit information from directors only. Although it is more costly and logistically more complicated, future studies could consider also collecting information from classroom teachers.

When asked if lack of support from teachers or administrators was a barrier to promoting healthy eating and physical activity, few directors or teachers said yes. This suggests that support for obesity prevention programs exists at multiple levels in Head Start centers, which could account for a potential link between attending Head Start and better preschool weight status reported in a recent study (17). Overall, teachers reported more barriers to promoting healthy eating than directors, suggesting that teachers may need more support to effectively encourage healthy eating. The highest reported barriers to promoting healthy eating according to teachers were limited time, insufficient funds, and lack of support from food service staff. A previous study of Head Start directors reported similar barriers. In that study, insufficient funds (51%) was the highest barrier to implementation of nutrition programs, followed by lack of support by food service providers (25%) and limited time (22%) (5), which suggests that these barriers may be common in Head Start centers. Future interventions should consider these barriers in program development and implementation.

This study had limitations. The sample was restricted to recruited Head Start centers in Austin and Houston, Texas, which may limit our ability to generalize the results to ECE centers that do not participate in Head Start or to centers outside of these cities. Given the self-reported nature of the survey, director and teacher responses were based on their perceptions of best practices and barriers, which may not reflect the actual ECE center environment. Self-reported data are subject to response bias, and social desirability bias may result in the directors and teachers overstating best practices and downplaying barriers. Considering that directors were more likely to report best practices and less likely to report barriers, the social desirability bias may affect directors more, which highlights the need to survey both teachers and directors when assessing the ECE center. Strengths of this study include the use of validated and widely used scales (NAP SACC and the Child Care Nutrition and Physical Activity Assessment Survey) to assess the practices and perceived barriers that enable comparison with similar studies in the United States, though the questions were modified in 2 ways. First, to determine the different barriers faced for promoting healthy eating and physical activity, we adapted the original Child Care Nutrition and Physical Activity Assessment Survey (11) to ask about barriers per subject separately. Second, in the original validation study (11) for the Child Care Nutrition and Physical Activity Assessment Survey, the surveys were completed by center directors, not classroom teachers as in our study. Future validation studies are needed to ensure the validity of the modified instruments.

This study was one of the first to compare Head Start teachers’ and directors’ perceptions of the implementation of best practices related to promoting healthy eating and physical activity and barriers to their implementation in their ECE programs. Because teachers are the direct caregivers responsible for implementation of policies, programs, and practices in the classroom, it is important to assess their perceptions of barriers and best practices rather than to rely solely on responses from directors. Future research should continue to use validated instruments to assess perceptions of teachers or primary caregivers as they relate to promoting healthy eating and physical activity.

## References

[1] Lumeng JC , Kaciroti N , Sturza J , Krusky AM , Miller AL , Peterson KE , Changes in body mass index associated with Head Start participation. Pediatrics 2015;135(2):e449–56. 10.1542/peds.2014-1725 25583912 PMC4306793

[2] US Department of Health and Human Services, Administration for Children and Families, Office of Head Start. Report to Congress on Head Start efforts to prevent and reduce obesity in children. https://eclkc.ohs.acf.hhs.gov/sites/default/files/pdf/head-start-efforts-prevent-reduce-obesity-children.pdf. Accessed Mary 23, 2017.

[3] Head Start program facts fiscal year 2015. Head Start, Office of the Administration for Children and Families, Early Childhood Learning & Knowledge Center, 2015. https://eclkc.ohs.acf.hhs.gov/sites/default/files/pdf/head-start-fact-sheet-fy-2015.pdf. Accessed May 23, 2017.

[4] Ling J , Robbins LB , Wen F . Interventions to prevent and manage overweight or obesity in preschool children: a systematic review. Int J Nurs Stud 2016;53:270–89. 10.1016/j.ijnurstu.2015.10.017 26582470

[5] Hughes CC , Gooze RA , Finkelstein DM , Whitaker RC . Barriers to obesity prevention in Head Start. Health Aff (Millwood) 2010;29(3):454–62. 10.1377/hlthaff.2009.0499 20194987

[6] Nanney MS , LaRowe TL , Davey C , Frost N , Arcan C , O’Meara J . Obesity prevention in early child care settings. Health Educ Behav 2017;44(1):23–31. 10.1177/1090198116643912 PMC5563467 27198534

[7] Tandon PS , Walters KM , Igoe BM , Payne EC , Johnson DB . Physical activity practices, policies and environments in Washington State child care settings: results of a statewide survey. Matern Child Health J 2017;21(3):571–82. 10.1007/s10995-016-2141-7 27449654 PMC5253339

[8] Hoelscher DM , Butte NF , Barlow S , Vandewater EA , Sharma SV , Huang T , Incorporating primary and secondary prevention approaches to address childhood obesity prevention and treatment in a low-income, ethnically diverse population: study design and demographic data from the Texas Childhood Obesity Research Demonstration (TX CORD) study. Child Obes 2015;11(1):71–91. 10.1089/chi.2014.0084 25555188 PMC4696423

[9] Sharma S , Chuang R-J , Hedberg AM . Pilot-testing CATCH Early Childhood: a preschool-based healthy nutrition and physical activity program. Am J Health Educ 2011;42(1):12–23. 10.1080/19325037.2011.10599169

[10] Ward DS , Benjamin SE , Ammerman AS , Ball SC , Neelon BH , Bangdiwala SI . Nutrition and physical activity in child care: results from an environmental intervention. Am J Prev Med 2008;35(4):352–6. 10.1016/j.amepre.2008.06.030 18701236

[11] Henderson KE , Grode GM , Middleton AE , Kenney EL , Falbe J , Schwartz MB . Validity of a measure to assess the child-care nutrition and physical activity environment. J Am Diet Assoc 2011;111(9):1306–13. 10.1016/j.jada.2011.06.011 21872693 PMC4172575

[12] Ward DS , Mazzucca S , McWilliams C , Hales D . Use of the Environment and Policy Evaluation and Observation as a Self-Report Instrument (EPAO-SR) to measure nutrition and physical activity environments in child care settings: validity and reliability evidence. Int J Behav Nutr Phys Act 2015;12(1):124. 10.1186/s12966-015-0287-0 26410387 PMC4583722

[13] Reilly JJ . Low levels of objectively measured physical activity in preschoolers in child care. Med Sci Sports Exerc 2010;42(3):502–7. 10.1249/MSS.0b013e3181cea100 20068499

[14] Ward S , Bélanger M , Donovan D , Carrier N . Systematic review of the relationship between childcare educators’ practices and preschoolers’ physical activity and eating behaviours. Obes Rev 2015;16(12):1055–70. 10.1111/obr.12315 26345462

[15] Edelson LR , Mokdad C , Martin N . Prompts to eat novel and familiar fruits and vegetables in families with 1–3 year-old children: Relationships with food acceptance and intake. Appetite 2016;99:138–48. 10.1016/j.appet.2016.01.015 26792770

[16] Sisson SB , Campbell JE , May KB , Brittain DR , Monroe LA , Guss SH , Assessment of food, nutrition, and physical activity practices in Oklahoma child-care centers. J Acad Nutr Diet 2012;112(8):1230–40. 10.1016/j.jand.2012.05.009 22818731

[17] Swyden K , Sisson SB , Lora K , Castle S , Copeland KA . Association of childcare arrangement with overweight and obesity in preschool-aged children: a narrative review of literature. Int J Obes 2017;41(1):1–12. 10.1038/ijo.2016.198 27811950

